# An Inspiring Journey of Hope and Persistence: Life Lessons with Françoise Barré-Sinoussi

**DOI:** 10.3390/v14051108

**Published:** 2022-05-21

**Authors:** Kiho Tanaka, Paula M. Cevaal, Rachel D. Pascoe

**Affiliations:** 1 Department of Infectious Diseases, The University of Melbourne at The Peter Doherty Institute for Infection and Immunity, Melbourne, VIC 3000, Australia; ktanaka@student.unimelb.edu.au; 2 Department of Microbiology and Immunology, The University of Melbourne at The Peter Doherty Institute for Infection and Immunity, Melbourne, VIC 3000, Australia; pmcevaal@student.unimelb.edu.au

Three early-career female virologists sat down with a distinguished Nobel laureate to discuss two pandemics, 39 years apart.

## 1. Preface

In an era where a novel coronavirus continues to dominate the news, we spoke with a celebrated virologist that devoted her life to another virus. In 1982, when clinicians were grappling with the early days of the AIDS pandemic, Françoise Barré-Sinoussi made the Nobel prize-winning discovery of the Human Immunodeficiency Virus (HIV) as the causative agent of AIDS [1]. We asked Françoise about her early career, the challenges she faced, the drivers behind her enduring passion and her view on the COVID-19 pandemic response. 

## 2. Establishing Her Research Career

When asked about the start of her scientific journey, she says she was “a bit of a peculiar case”. Unsure of whether she was going in the right direction after two years of undergraduate study, Françoise approached many labs to volunteer to “understand better what it meant to be a researcher”. Despite this enthusiasm, she was only offered a volunteer position in two laboratories. As luck would have it, one of these research projects put her on the path of a lifelong passion. Françoise joined Jean-Claude Chermann’s laboratory in 1971, focusing on retroviruses and cancer—one year after the discovery of the reverse transcriptase enzyme by David Baltimore and Howard Temin [2,3]. At the time, there were no known human retroviruses; the first, HTLV, was discovered by Robert Gallo and colleagues in 1979 [4]. Françoise was awarded her Ph.D. degree in 1974 and moved to the National Institute of Health (NIH) for her post-doctoral degree, before returning to the Institut Pasteur with the Chermann laboratory, under the departmental head Professor Luc Montagnier.

Françoise was working on retroviruses when the first reports of the AIDS epidemic in France emerged. “At that time, we did not realize the magnitude of the epidemic at all. We were in early ‘80s in my country, in France, and if I remember we had around 100 patients. It wasn’t until later that we found out what was going on, for example, in Africa”, Françoise reflects. She recounts feeling sceptical when first approached by Willy Rozenbaum and his team of clinicians to investigate the causative agent of AIDS. “They had in mind that HTLV could be the cause of AIDS, but we immediately told them we did not believe this hypothesis. [HTLV] was known to immortalize T cells, yet clinicians were reporting to us that the patients were *losing* their CD4 lymphocytes”. Françoise’s post-doctoral research experience at the Institut Pasteur on non-human retroviruses associated with immune deficiency influenced her decision to investigate retroviruses, while holding reservations that HTLV was the causative agent of AIDS. The team performed reverse transcriptase assays on a lymphocyte culture from a lymph node biopsy of a patient with pre-AIDS, “**it was worth testing the hypothesis**”. An example of outstanding collaboration between scientists and clinicians – their strategic choice of patient sample to test the hypothesis proved key for isolating and detecting the virus at the time.

Françoise soon confirmed that HTLV was indeed *not* the virus detected, suggesting they had identified a novel human retrovirus as the probable cause of AIDS. What followed was an astonishing conversation with her mentor in the US, who advised her to throw her work to the garbage. “You’re completely crazy”, she remembers thinking during that phone call. Reflecting on it, Françoise explains he must have foreseen the politics that would soon surround their discovery. It took a good deal of further validation experiments before they were confident in their findings; testing samples from multiple individuals with AIDS, using electron microscopy to visualise the virus, immunoprecipitation to confirm no cross-reactivity between the virus and anti-HTLV monoclonal antibodies, and performing large seroepidemiological studies to establish the etiological link between the virus and the disease.

“By then, it was February 1983, so we were rushing quite a lot. But I do remember one night we had dinner at the student base, and we definitely celebrated that night!”.

Despite discovering HIV, Françoise has experienced similar challenges in academia as many other scientists. In particular, she discusses her challenges in establishing her own lab. Between 1988 and 1992, Françoise failed to receive support from her department, until an international review committee highlighted her success during a site visit of the Virology Department of the Institut Pasteur. “You [need to be] recognised, not only at national level but at an international level. It is the best way to convince those who are not fully convinced because you are a female to make the decision. In addition, my relationship with my former boss was far from optimal, which was clearly an obstacle for me”. Being a woman in science, Françoise comments on the challenges faced to be viewed as an accomplished scientist, rather than an accomplished female scientist. She believes that gender should play no part in hiring or recognition of scientific accomplishments: “**Female scientists should be viewed as successful according to their scientific quality, not because they are female scientists**. I say to many of my students, female in particular, when you want, you can”.

## 3. Beyond the Lab

Françoise’s work goes beyond the lab. She highlights how her extensive outreach and community involvement has shaped her profoundly: “My contact and my relationship with people living with HIV have been my motivation since the very beginning when I started to work on this disease”. Upon reflecting on her initial interactions with people with HIV or AIDS, Françoise recalls being sought out by patients: “I did not even have to ask to be in contact with patients. Patients were coming to the Institut Pasteur because they were HIV positive, they were dying from this virus, and they were coming to see us, to see me, not a clinician. I was somehow obliged from the beginning to be with them. They wanted to know what we were going to do to treat them”.

These interactions with people with HIV and AIDS proved a turning point in her career. “I realised how much it was important also for me, to better understand their expectations, what they were expecting from us as scientists. Additionally, **each time they were telling me they want a treatment they can stop. It is our duty as a scientist to make it**”. Françoise has been involved in the development and leadership of many organisations and international societies, including AIDES, an AIDS non-governmental organization in France (established in 1984). She coordinated the French National Agency for Research on AIDS and Viral Hepatitis (ANRS) research programs in Cambodia and Vietnam and was President of the International AIDS Society (IAS). Françoise has been Director of Research at the National Institute for Health and Medical Research (INSERM), Head of the Retroviral Infection Unit at the Institut Pasteur and she is still Honorary President of the Virology Department and of the Pasteur Network. As part of her role as President of the IAS, Françoise launched the IAS “Towards a HIV Cure” Initiative in 2010. “I have seen a lot of progress since that time - all research was very dispersed, everywhere in the world. I’ve seen a lot of consortia that started with people working together, which is the best way to do it if we want to make progress”.

When asked whether an HIV cure will be possible, Françoise’s response is very direct and honest: “**Personally, I do not believe we will have a cure**. I strongly believe that we can have a treatment that will induce sustainable remission and that it will be a combination of several strategies. There is a lot of work going on and I think we are progressing in the right direction… it just takes time”. She highlights the importance of the ongoing research that investigates the mechanism of HIV persistence. This research will be crucial to understand how to best approach sustainable remission and tackle why individuals respond differently to treatments—to collectively fill in the gaps of the HIV puzzle. “It seems like it’s going in all directions today. At the point where we are in science, that’s quite normal. But at one point everything will be clarified”.

In her work with many local communities, Françoise has witnessed the evolution of attitudes towards HIV/AIDS. She feels the perceptions of society were slow to change but things are now changing, despite the politics. The unstable political situations in countries such as the Central African Republic in the ‘80s posed challenges, she tells us, “During the first five years, it was a positive evolution among the population and the clinicians, then it became a political issue. The political situation clearly impacts the evolution of countries”. Françoise also worked in Cameroon, where minority populations are still experiencing physical violence and marginalisation. She took it upon herself to raise the problem with the First Lady of Cameroon, who explained that these issues involved the country’s traditions and conservative views which are difficult to challenge. Françoise said in return, "Look, we have traditions everywhere. But at one point you must initiate an evolution and it will take time because that reflects the education of the population". On the brighter side, Françoise feels that attitudes in Cambodia and Vietnam are now evolving.

## 4. Stigma and Sex Education 

A key factor that drives the politicisation of HIV is the taboo around sexually transmitted infections, reflecting a larger taboo on topics regarding sex and sex workers in many cultures. Growing up, Françoise did not regard sexuality and sex industry as taboos, but they were not openly discussed. However, as she progressed in her career, Françoise realised and truly felt the stigmatisation and discrimination of certain populations in her country. She could not believe the lack of understanding in the public, as diversity and tolerance were important values in her education. “Enough is enough, we have to work together in order to fight this kind of situation”, she remembers thinking. To improve the sexual health literacy of our young population, Françoise highlights the importance of good quality sex education being part of secondary school curricula, not just at universities, as well as the role of parents in their children’s education. Françoise holds that there has been some advancement in sex education compared to the early ‘80s, however, certain information has not been updated at all. “Some [teenagers] still think that HIV can be transmitted by mosquitoes. Some of them think that there is a cure for HIV. So, you realize that really what they are learning at school is at a poor standard.” Furthermore, many of these conversations are not happening in the classroom or at home, but are conversations between friends, as Françoise recounts from her own experience. It takes several generations to make the uncomfortable comfortable, “because if we educate now, the young population will be more open about sex, homosexuality, transgender individuals etc., then that will be part of their life, part of their mind.” Within the next ten or twenty years, Françoise feels confident that we will be able to see some positive evolution in the knowledge and perceptions towards HIV. With accurate, evidence-based information taught and accepted in the community, we can reduce both HIV stigma and discrimination.

## 5. HIV and COVID-19

Having worked at the forefront of the HIV/AIDS pandemic both in the lab and outside, Françoise is able to offer a unique perspective on the research strategy during the COVID-19 pandemic: “The strategy was the same. The timeline is not the same. The research has been much more rapid for SARS-CoV-2 than for HIV because of the wonderful progress in technology. In the 1980’s, PCR did not exist. How could we clone and sequence a virus?”. Françoise also highlights how the societal approach to the pandemics differed, “One thing missing for SARS-CoV-2 is there was not enough involvement of the civil society. **One thing we have learnt from HIV is how important it is to work with representatives of patients**”. According to Françoise, this was not done well with COVID-19, hindering effective communication and implementation of public health regulations, negatively impacting the rapport between health officials and the community, and contributed to the public mistrust and vaccine hesitancy that was seen globally.

## 6. Message to Us

Twenty-five years after the discovery of HIV in 1983, Françoise received the 2008 Nobel Prize in Physiology or Medicine, alongside her co-researcher Luc Montagnier, for their discovery of HIV as the causative agent of AIDS [5]. The Nobel Prize was shared with Harald zur Hausen, who discovered Human Papilloma Virus (HPV) as the causative agent of cervical cancer. Françoise should not be solely defined by her Nobel Prize, but instead acknowledged for her continuing contribution to the HIV pandemic, helping educate the community, advocating for people living with HIV and being a leader in HIV research. Françoise has spent nearly half a century dedicating her life to people living with HIV. In the spirit of this Virology: Women in Science edition, we asked Françoise what message she would give early-career female researchers. What she had to offer was a message that will resonate with a much wider community: “Be as persistent as HIV is”.
